# Oxidative Stress and Inflammation in Renal Patients and Healthy Subjects

**DOI:** 10.1371/journal.pone.0022360

**Published:** 2011-07-28

**Authors:** Diana M. Lee, Kenneth W. Jackson, Nicholas Knowlton, Joshua Wages, Petar Alaupovic, Ola Samuelsson, Aso Saeed, Michael Centola, Per-Ola Attman

**Affiliations:** 1 Lipid and Lipoprotein Studies, Oklahoma Medical Research Foundation, Oklahoma City, Oklahoma, United States of America; 2 Molecular Biology-Protemics Facility, University of Oklahoma Health Sciences Center, Oklahoma City, Oklahoma, United States of America; 3 Microarray Analysis and Proteomics Core, Oklahoma Medical Research Foundation, Oklahoma City, Oklahoma, United States of America; 4 Department of Nephrology, Sahlgrenska University Hospital, University of Göteborg, Göteborg, Sweden; New York University, United States of America

## Abstract

The first goal of this study was to measure the oxidative stress (OS) and relate it to lipoprotein variables in 35 renal patients before dialysis (CKD), 37 on hemodialysis (HD) and 63 healthy subjects. The method for OS was based on the ratio of cholesteryl esters (CE) containing C18/C16 fatty acids (R2) measured by gas chromatography (GC) which is a simple, direct, rapid and reliable procedure. The second goal was to investigate and identify a triacylglycerol peak on GC, referred to as TG48 (48 represents the sum of the three fatty acids carbon chain lengths) which was markedly increased in renal patients compared to healthy controls. We measured TG48 in patients and controls. Mass spectrometry (MS) and MS twice in tandem were used to analyze the fatty acid composition of TG48. MS showed that TG48 was abundant in saturated fatty acids (SFAs) that were known for their pro-inflammatory property. TG48 was significantly and inversely correlated with OS. Renal patients were characterized by higher OS and inflammation than healthy subjects. Inflammation correlated strongly with TG, VLDL-cholesterol, apolipoprotein (apo) C-III and apoC-III bound to apoB-containing lipoproteins, but not with either total cholesterol or LDL-cholesterol.

In conclusion, we have discovered a new inflammatory factor, TG48. It is characterized with TG rich in saturated fatty acids. Renal patients have increased TG48 than healthy controls.

## Introduction

A progressive loss of renal function is associated with a markedly increased risk for cardiovascular disease (CVD) [1]. Among non-traditional risk factors, increased oxidative stress (OS) and inflammation have emerged as potentially significant contributors to the progression of kidney dysfunction and its cardiovascular consequences. Although a number of studies have shown increased OS and inflammation in renal patients [2]–[12], no publication has demonstrated the relationship between OS/inflammation and plasma apolipoproteins (apo) and lipoproteins (Lp), including the well established risk factors for CVD, such as triacylglycerols (TG) and apoC-III. Since the relationship between OS/inflammation and CVD in renal patients is not well understood, the first aim of this study was to investigate the possible relationship between OS/inflammation and lipoprotein variables already known to be related to CVD with special emphasis on apoC-III, which has recently been shown to be a link between inflammation and atherogenesis [13]. The present study was performed on patients undergoing haemodialysis (HD) and on patients before dialysis (CKD), using a simple cholesteryl ester (CE) ratio method on gas chromatography (GC) for OS [14]. Interestingly, during the measurement of OS with GC, we found marked differences between renal patients and healthy subjects regarding a TG species, named TG48 (i.e. the sum of the 3 carbon chain lengths of fatty acids in TG equals to 48) which was absent or undetectable in healthy young normolipidemic subjects but markedly increased in renal patients. We wondered what the significance of TG48 is in renal disease and what types of fatty acids are present in TG48. Thus, the second aim of this study was to investigate the TG48, i.e. to measure TG48 with GC in renal patients and healthy subjects, and to identify the TG48 components using mass spectrometry (MS) and MS/MS.

## Materials and Methods

### Patients and Controls

Seventy two non-diabetic adult patients (46 men and 26 women) participated in the study. The patients' mean age was 61±13 (range 27–84) years. Patients were recruited from the Department of Nephrology, Sahlgrenska University Hospital, Göteborg, Sweden, and collaborating hospitals in Western Sweden.

There were 35 patients (23 men and 12 women) with varying degrees of renal functional impairment before dialysis (CKD stages 3–5). In these patients, the mean glomerular filtration rate (GFR) was 26.3 mL/min/1.73 BSA (body surface area), with a range from 9 to 53. Patients were treated with antihypertensive agents, angiotensin converting enzyme (ACE) and by diuretics and phosphate binders, as appropriate. A number of patients with severely reduced renal function (GFR <20 mL/min) were advised to consume a protein reduced diet (0.6–0.8 g protein/kg BW/24 h) for the treatment of symptoms of renal insufficiency.

Thirty seven patients (23 men and 14 women) were treated with chronic haemodialysis (HD). Haemodialysis was performed either with conventional haemodialysis or with haemodiafiltration using standard procedures and equipment. All patients were dialyzed with bicarbonate-containing dialysis fluid. In the majority of cases low molecular weight heparin was added as an anticoagulant. Patients were treated three times weekly for four to five hours per session with antihypertensive drugs, diuretics, calcium supplements, phosphate binders, intravenous iron and erythropoietin, as appropriate. The target hemoglobin levels were 120–130 g/L.

Patients on steroid treatment, lipid-lowering drugs or other drugs that may have influenced lipoprotein metabolism, were excluded from the study as were patients with diabetes mellitus. Patients were not advised or monitored with respect to dietary lipids. None of the patients received intravenous iron, vitamin D or intravascular radiocontrast media within a week before blood sampling. Patients were not specifically treated with larger doses of vitamin E preparations.

Control subjects were recruited among healthy, normolipidemic employees (30 men and 33 women) of the Sahlgrenska University Hospital, Göteborg, Sweden and Oklahoma Medical Research Foundation, Oklahoma City, Oklahoma, USA. All studies were conducted in accordance with the Helsinki declaration and approved by the Ethical Committee of the Sahlgrenska University Hospital, Göteborg, Sweden. All subjects gave their written informed consent.

Blood samples were drawn after an over-night fast and collected into EDTA-containing tubes. In HD patients, blood was drawn prior to heparinization for dialysis through the dialysis access. Blood samples were then centrifuged. Preservatives containing thimerosal and a protease inhibitor, ε-amino caproic acid, were added to a final concentration of 0.1% and 10 mM respectively, and immediately shipped by air to Oklahoma City, Oklahoma, USA, where analyses were performed. Glomerular filtration rate was measured by renal or plasma clearance of either iohexol or ^51^Cr-EDTA, as previously described [15].

### Lipid and apolipoprotein analyses

Plasma TC (Alfa Wassermann; Caldwell, NJ)) and TG (Alfa Wassermann; Caldwell, NJ) concentrations were determined enzymatically on Alfa Wassermann Nexct Analyzer. High-density lipoprotein-cholesterol (HDL-C) was measured as previously described [15]. Very low density lipoprotein cholesterol (VLDL-C) levels were assumed to equal one fifth of the plasma TG concentration, and low density lipoprotein-cholesterol (LDL-C) levels were determined as described previously [15], [16].

The quantitative determination of apolipoproteins (apo) was performed by electroimmunoassays developed in our laboratory [15], [16]. The measurement of apoC-III bound to apoA-I (HDL) and apoC-III bound to apoB-(VLDL+LDL)-containing lipoproteins was performed on heparin-Mn^++^ supernates (apoC-III-HS) and heparin-Mn^++^ precipitate (apoC-III-HP), respectively, according to a previously described procedure [15], [16]. The apoC-III-ratio (apoC-III-R) was calculated as apoC-III-HS/apoC-III-HP.

### Measurement of oxidative stress by cholesteryl ester ratios method

Indices of OS were measured according to the method developed by Lee [14]. Briefly, the neutral lipids were extracted from fresh plasma with isopropanol:heptane (7∶3) containing internal standard (I.S.) CE-butyrate (CE-C4) under argon. After acidification with dilute H_2_SO_4_, the top heptane phase was concentrated under N_2_, and the neutral lipid molecular species were analyzed by GC as described by Kuksis et al [17] and modified by Lee [14], using Varian Chrompack CP-3000 Gas Chromatograph and ultra-pure helium gas as carrier for vaporized samples. The column was CP 7592, WCOT Ulti-metal, coating HT Simdist film thinness 0.52 µm, 10 m in length and 0.53 mm in diameter. The initial temperature of the column, 160°C, was increased to 380°C at a rate of 10°C per minute. The area on the GC chromatogram for CE containing C16 fatty acyl groups was calibrated with recrystalized cholesteryl palmitate; CE containing C18 fatty acyl groups was calibrated with cholesteryl linoleate and CE containing C20 fatty acyl groups with cholesteryl arachidonate. All primary standards were from Nu-Chek-Prep, Inc. Elysian, MN, USA.

Cholesteryl esters with C16 fatty acyl groups are mostly saturated fatty acids (C16:0) with a small amount of monounsaturated fatty acid (C16:1), which is considered not susceptible to oxidative damage. Cholesteryl esters with C18 fatty acyl groups are mostly C18:2 with small amounts of C18:1 and C18:0. The C18:1 and C18:0 are resistant to oxidation while CE-C18:2 are susceptible to oxidative damage. Cholesteryl esters with C20 fatty acyl groups are mostly C20:3 with small amounts of C20:4 and C20:5, which are the most sensitive to oxidative damage. Since it is possible to separate the molecular species of CE by the number of carbon atoms of esterified fatty acids, it is possible to calculate the ratios CE-C20/CE-C16 (R1) and CE-C18/CE-C16 (R2) as estimates of peroxidative damage [14]. Due to a greater loss of polyunsaturated fatty acid esters to oxidative damage, lower CE ratios indicate a higher OS. The method was verified with thiobarbituric acid-reactive substances (TBARS) since both R1 and R2 are negatively correlated with TBARS (r = −0.68, p<0.0001, n = 24, for R1, and r = −0.62, p<0.001, n = 25, for R2) [14]. Since the sizes of CE-C18 peaks are greater than those of CE-C20, the measurement of the former are more accurate than those of the latter. Therefore, we have selected R2 as the marker for OS in this study.

### Measurement of TG species with gas chromatography

From the same above neutral lipid extract, TG species were measured following CEs separations. TG48 was calibrated with tripalmitate. TG54 was calibrated with tristearate. (All primary standards were from Nu-Chek-Prep, Inc. Elysian, MN). Two reference sera (from Boehringer-Mannheim Corp., Indianapolis, IN) with known TC and TG values were also used to verify the TC and the TG values derived from the sum of molecular species of neutral lipids by GC. TG species include TG48, TG50, TG52, TG54 and TG56 (the numbers following TG represent the sum of the 3 carbon chain lengths of fatty acids). Occasionally, CE-C20 and TG48 did not separate completely. A vertical line drawing through the valley of the two peaks was used for separation and measurements. These measurements were validated by re-application with smaller amount of samples that could be separated completely.

The GC method yielded within 97–99% of known TC values and 95–106% of known TG values. The intra-assay variations were 3.34% for R2, 2.24% for CE, 5.64% for TG and 4.22% for TG48. The inter-assay variations were 0.90% for R2, 4.19% for CE, 5.3% for TG and 5.94% for TG48.

### Measurement of TG48 with GC and identification of the components of TG48 by Mass Spectrometry

**Figure 1:**
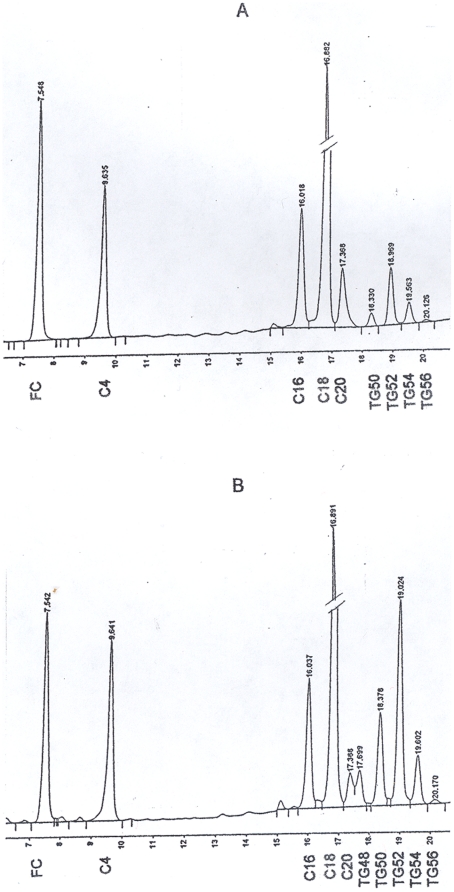
GC chromatograms. **Fig. 1A. A typical GC chromatogram of separation of neutral lipid molecular species from a normal subject.** The separated FC, CE molecular species with C16, C18 and C20 fatty acyl groups and TG molecular species termed TG50, TG52, TG54 and TG56 (the numbers represent the sum of the carbon chain lengths of the three fatty acyl groups of TG) are identified at the bottom of each peak. Note the absence or undetectable of the peak TG48. **Fig. 1B. An example of the GC chromatogram of separation of neutral lipid molecular species from a HD patient.** The separated molecular species are identified at the bottom of each peak. Note the presence of the peak TG48 at retention time 17.699 min. in this renal patient. In case when TG48 was not completely separated from CE-C20, re-run with a reduced sample application was carried out and complete separation could be achieved. Or, a vertical line drawing through the valley as this one, the results were close to the former method.

For example, as demonstrated in Fig. 1A a GC chromatogram of a young fasting healthy subject with low plasma TG showed the absence of TG48 peak. However, in renal patients the TG48 peak height increased (See example in Fig. 1B). For identification of compounds occurring in TG48 peak, MS was used, following the method of Liebisch et al. [19]. The same hexane extract (10 µl) of plasma neutral lipids containing I.S. CE-C4 for GC analyses was evaporated under a stream of nitrogen. The sample was then redissolved in 50 µl of a solvent mixture of methanol and chloroform (3∶1 v:v) containing 7.5 mM ammonium acetate. This solution was then analyzed by direct flow injection analysis using a syringe pump at a flow rate of 5 µl/min. Bruker Daltonics Inc. HCT ultra ion-trap mass spectrometer equipped with an electrospray ion source was used and operated in the positive ion mode. Samples ionized in the presence of ammonium acetate carried an NH_4_^+1^ ion, so the mass/charge (m/z) increased by 18 over that of the uncharged molecule. CE-C4 with +1 charge was confirmed to have m/z 474.3 on MS. The expected m/z for saturated TG48:0, monounsaturated TG48:1, di-unsaturated TG48:2 and tri-unsaturated TG48:3 are shown in Table 1. Standard tripalmitate (TG48:0 from Nu-Chek-Prep) with +1 charge was confirmed to have m/z = 824.7. Standard tripalmitolein (TG48:3 from Nu-Chek-Prep.) with +1 charge was confirmed to have m/z = 818.7. The areas of all m/z peaks observed within the measured mass range were calculated automatically by the instrument software. After normalizing the areas of I.S. from all runs, the intensities from different runs could be compared.

**Table 1:** Calculated molecular mass and m/z of saturated and unsaturated TG48.

Names of compounds	Symbols	Mass	m/z with NH_4_^+1^
Cholesterol butyrate (I.S.)	CE-C4	456.3	474.3
Tripalmitin (tripalmitylglycerol)	TG48:0	806.74	824.71
1,3-Palmityl-2-palmitoleylglycerol	TG48:1	804.74	822.74
Dipalmitoleyl-palmitylglycerol	TG48:2	802.74	820.74
Tripalmitoleylglycerol	TG48:3	800.74	818.74

### Identification of the fatty acids composed in TG48 by MS/MS

The fatty acid composition of TG48 was determined by MS/MS of ammoniated triacylglycerol (TG) ions isolated in the first stage MS. The MS/MS data was obtained by collisional induced disassociation at 3.5 volts of selected TG ions, essentially according to the method of McAnoy et al. [20]. Ammoniated TG (parent) ions at 818.6, 820.7, 822.7, and 824.7 m/z corresponding to the m/z values of TG48:3, TG48:2, TG48:1 and TG48:0, respectively, were individually fragmented, generating TG ^+1^ as a result of loss of ammonia, diacylglycerols (DG) and DG fragments at position 1,2; 2,3; and 1,3 for each TG fragmented. The fatty acids for each TG were identified by comparison of our products to the published m/z values for TG ^+1^, DG_1,2_, DG_2,3_, and DG_1,3_, [21]. When all DG and DG fragments observed by MS/MS were accounted for, the composition of fatty acids for each TG48 was considered completed.

### Statistical methods

A two-tailed T-test with Satterthwaite adjustment was used to identify whether there was a difference in means. A Pearson product moment correlation was used to quantitate the relationship between variables. Multiple linear regression was performed to create a multivariate model to predict TG48 concentrations. Data were analyzed in SAS v.9.1.3 (Cary, NC) and R v.2.5.1 (Vienna, Austria). Univariate p-values were adjusted for multiplicity through the Benjamini & Hochberg False Discovery Rate (FDR) procedure [22]. A 5% FDR was considered statistically significant.

## Results

Due to its high correlation with an established OS marker, TBARS [14], R2 was used as a valuable index of OS. The R2 and TG48 levels for patients with CKD before dialysis and patients on chronic maintenance haemodialysis (HD) compared to those of normal controls are shown in Table 2. These data showed that the R2 in CKD and HD patients were significantly lower than that of healthy controls, thus higher OS. The data also showed that TG48 in CKD and HD patients were significantly higher than that of healthy controls. Pearson correlations were calculated between TG48 and R2 for each of the three groups: CKD, HD and controls. CKD (r = −0.39, p-value  = 0.02), and HD (r = −0.41, p-value  = 0.01) both had significant p-values associated with their correlations. In contrast, controls had a non-significant p-value (r = -0.17, p-value  = 0.18) which is understandable, since a large number of controls had zero or near zero TG48 values. These results indicated that TG48 levels were closely related to OS in CKD and HD patients.

**Table 2:** Oxidative stress R2, lipids and apolipoproteins in patients with kidney disease compared to controls.

	Controls	CKD	HD
	n = 63	n = 35	n = 37
	Mean ± SD	Mean ± SD	Mean ± SD
R2	5.18±1.26	4.18±0.77^a^	4.48±0.68^b^
TG48 (mg/dL)	1.67±3.26	9.19±8.03^a^	5.29±3.48^a^
TC (mg/dL)	179.40±34.40	238.00±68.30^a^	199.10±48.30^e^
VLDL-C (mg/dL)	16.80±6.30	32.60±17.40^a^	26.40±11.20^a^
LDL-C (mg/dL)	114.80±30.20	162.30±65.00^a^	133.00±43.00^e^
HDL-C (mg/dL)	48.30±12.70	42.00±11.30^d^	39.80±11.30^c^
TG (mg/dL)	85.00±31.40	161.50±83.10^a^	131.80±56.20^a^
ApoB (mg/dL)	91.40±20.20	136.10±50.20^a^	115.10±32.80^a^
ApoE (mg/dL)	6.32±1.79	10.65±3.93^a^	8.53±2.49^a^
ApoC-III (mg/dL)	9.06±2.25	17.91±7.52^a^	16.83±6.42^a^
ApoC-III-HS (mg/dL)	6.01±4.50	7.15±3.90	10.02±4.82^a^
ApoC-III-HP (mg/dL)	3.89±3.74	9.01±5.42^a^	5.76±2.79^d^
ApoC-III-R	1.84±0.97	1.21±1.16^d^	2.15±1.30
Age (years)	44.30±13.10	55.10±12.50^a^	65.90±10.60^a^
Gender (Males)	30 (48%)	23 (65%)	23 (62%)
Gender (Females)	33 (52%)	12 (35%)	14 (38%)

**Note:**

CKD  =  chronic kidney disease before dialysis; HD  =  patients with kidney disease on haemodialysis; TG  =  triglycerides; VLDL-C  =  very low density lipoprotein-cholesterol; TC  =  total cholesterol;

LDL-C  =  low density lipoprotein-cholesterol; HDL-C  =  high density lipoprotein-cholesterol;

ApoC-III-HS  =  apoC-III bound to HDL; ApoC-III-HP  =  apoC-III bound to apoB-containing lipoproteins;

ApoC-III-R  =  ratio of apoC-III-HS/apoC-III-HP.

Two-tailed T-test with Satterthwaite adjustment were used to test mean differences between controls and patient groups.

P-values reported are adjusted for multiplicity through the False Discovery Rate (FDR) procedure.

Superscripts denote: a, p<0.0005; b, p<0.001; c, p<0.005; d, p<0.025; e, p<0.05; no superscript = n.s.

Table 2 also shows the lipid and apolipoprotein profiles of renal patients and healthy controls.

The levels of TC, TG, VLDL-C, LDL-C, apoB, apoE, apoC-III, and apoC-III-HP were significantly higher, and HDL-C were significantly lower in CKD and HD compared to controls. The concentrations of apoC-III-HS were significantly higher in HD compared to controls, but not in CKD. The apoC-III-R was significantly lower in CKD, but not in HD.

The control group is younger than the patient population. However, analysis of the influence of age and gender shows that the contribution of age was very small as seen in TG48 model equation #1; therefore, this age difference is inconsequential to our conclusions. We may calculate the age effect by placing real numbers into this equation, assuming VLDL-C  = 33, an age difference of 11 years will affect only 5–6% of the TG48 value, while the value of TG48 in HD group is 317% higher, and TG48 in CKD group is 550% higher than controls.

BMI data was not listed in Table 2 because there were missing data in the HD and control groups. BMI for controls was 24.1±3.5 (n = 51) and for HD was 24.0±4.6 (n = 23), p =  not significant (N.S.)) when compared to controls. Therefore, the significant differences in R2 and TG48 between HD and control cannot be due to BMI effect. BMI for CKD was 25.7±3.6 (n = 35) (p<0.025) compared to controls.

There was no gender difference in the HD group for BMI, 24.5±4.51 (n = 7) for females, and 23.76±4.71 (n = 16) for males with p = N.S. The BMI for females and males in CKD group was 24.85±4.10 (n = 12), and 26.15±3.35 (n = 23) respectively with p =  N.S. The BMI for females and males in controls was 22.63±2.77 (n = 23) and 25.39±3.49 (n = 30) respectively with p<0.005 between females and males. There was, as expected, a correlation between BMI and TG, VLDL-C, apoC-III-HS, and HDL-C but no correlation between BMI and R2, TG48, GFR in CKD group.

Our data showed that there were no gender differences in R2 for CKD or for HD. Nor was there a gender difference in apoC-III-HP for CKD or HD.There was no correlation between GFR and R2 or TG48. GFR was negatively correlated with apoC-III, apoE, and apoC-III-HP and positively correlated with apoC-III-R. The lack of correlation between GFR and R2 or TG48 was probably due to narrow ranges of these values.

Correlation coefficients among TG48 and R2 and the lipoprotein variables of controls, CKD and HD are presented in Table 3. Among controls, TG48 moderately correlated with TG (r = 0.42, p<0.005), VLDL-C (r = 0.44, p<0.005), and weakly with apoC-III (r = 0.35, p<0.025) and inversely with apoC-III-R (r = −0.30, p<0.05). R2 was weakly correlated with apoC-III (r = -0.36, p<0.05) among all lipoprotein variables in controls.

**Table 3:** Correlations between oxidative stress marker R2, TG48 and lipid and lipoprotein variables.

	Controls	CKD	HD
	R2	R2	R2
	r (95% C.I.)	r (95% C.I.)	r (95% C.I.)
TC	−0.20 (−0.42, 0.05)	−0.17 (−0.48, 0.17)	0.23 (−0.11, 0.51)
VLDL-C	−0.06 (−0.31, 0.19)	−0.38 (−0.63, −0.05)	0.03 (−0.29, 0.35)
LDL-C	−0.15 (−0.38, 0.10)	−0.07 (−0.39, 0.27)	0.31 (−0.02, 0.58)
HDL-C	−0.24 (−0.46, 0.01)	0.05 (−0.29, 0.37)	−0.25 (−0.53, 0.09)
TG	−0.04 (−0.28, 0.21)^c^	−0.38 (−0.63, −0.05)	0.03 (−0.30, 0.35)
ApoB	−0.22 (−0.45, 0.03)	−0.17 (−0.48, 0.17)	0.12 (−0.21, 0.43)
ApoE	−0.26 (−0.48, −0.01)	−0.02 (−0.35, 0.32)	−0.02 (−0.34, 0.31)
ApoC-III	−0.36 (−0.56, −0.12)^e^	−0.41 (−0.65, −0.09)	0.04 (−0.29, 0.36)
ApoC-III-HS	0.14 (−0.12, 0.37)	−0.12 (−0.43, 0.22)	−0.04 (−0.36, 0.29)
ApoC-III-HP	0.15 (−0.10, 0.38)	−0.41 (−0.65, −0.09)	0.13 (−0.20, 0.44)
ApoC-III-R	−0.08 (−0.32, 0.18)	0.13 (−0.21, 0.44)	−0.11 (−0.42, 0.23)

	TG48	TG48	TG48
	r (95% C.I.)	r (95% C.I.)	r (95% C.I.)
TC	0.09 (−0.16, 0.33)	0.00 (−0.34, 0.33)	0.06 (−0.27, 0.37)
VLDL-C	0.44 (0.22, 0.62)^c^	0.78 (0.60, 0.88)^a^	0.47 (0.17, 0.69)^d^
LDL-C	0.08 (−0.17, 0.32)	−0.19 (−0.49, 0.15)	−0.06 (−0.38, 0.27)
HDL-C	−0.17 (−0.40, 0.08)	−0.25 (−0.54, 0.09)	0.01 (−0.31, 0.33)
TG	0.42 (0.19, 0.61)^c^	0.79 (0.62, 0.89)^a^	0.47 (0.17, 0.69)^d^
ApoB	0.15 (−0.10, 0.39)	0.16 (−0.18, 0.47)	0.11 (−0.22, 0.42)
ApoE	0.03 (−0.22, 0.27)	0.29 (−0.04, 0.57)	0.15 (−0.18, 0.45)
ApoC-III	0.35 (0.11, 0.55)^d^	0.49 (0.19, 0.71)^d^	0.04 (−0.29, 0.36)
ApoC-III-HS	−0.05 (−0.29, 0.20)	−0.03 (−0.36, 0.30)	−0.10 (−0.41, 0.23)
ApoC-III-HP	0.14 (−0.12, 0.37)	0.55 (0.26, 0.74)^c^	0.22 (−0.11, 0.51)
ApoC-III-R	−0.30 (−0.51, −0.06)^e^	−0.42 (−0.66, −0.11)^d^	−0.22 (−0.51, 0.11)

**Note:**

Abbreviations used here are the same as in Tables 2.

FDR adjusted p-values, significant differences:

a, p<0.0005; b, p<0.001; c, p<0.005; d, p<0.025; e, p<0.05; no superscript = n.s.

In CKD patients, TG48 was strongly positively correlated with TG (r = 0.79, p<0.0005), and VLDL-C (r = 0.78, p<0.0005), moderately correlated with apoC-III-HP (r = 0.55, p<0.005), apoC-III (r = 0.49, p<0.025) and inversely with apoC-III-R (r = - 0.42, p<0.025). No lipoprotein variables were significantly correlated with R2 in CKD patients (Table 3).

In HD patients, TG48 was moderately correlated with TG and VLDL-C (r = 0.47, p<0.025) (Table 3). Correlations between lipoprotein variables and R2 were not significant.

Multivariate linear regression modeling revealed that VLDL-C and LDL-C could be combined to predict TG48 in renal patients when adjusted for age. The data were modeled according to the following equation:
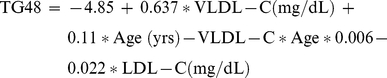
(1)


The model explained 61.5% of the variability in TG48. Examining the partial sums of squares revealed that VLDL-C explained 51% of the variability while LDL-C explained only 3.3% of the variability.

Alternatively, a second model showing apoC-III-HP and apoB with LDL-C could be combined to predict TG48 according to the following equation:
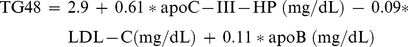
(2)This model explained 44.1% of variability in TG48. Examining the partial sum of squares revealed that apoC-III-HP explained 29.2% of the variability in the model. Thus, apoC-III bound to apoB-containing lipoproteins are most associated with TG48 among all lipoprotein particles. Since apoC-III-HP is a known pro-inflammatory factor [13], and there is a direct relationship between TG48 and apoC-III-HP, we can therefore conclude that there is relationship between TG48 and inflammation. Note that the equation #2 contains no age effect and no BMI effect.

### Identification of the Saturated and Unsaturated Forms of TG48 by MS

**Figure 2:**
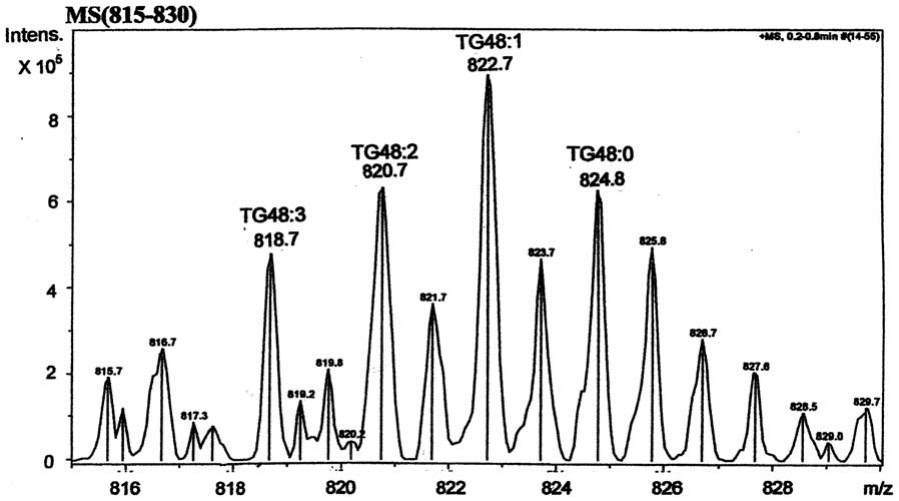
Mass spectrometry of TG48 species. Mass spectrometry of plasma neutral lipids of a patient was analyzed as described in the Methods. TG48 species were zoomed at 815–830 m/z. Peaks were identified based on their m/z values matched to the known compounds which were labeled on top or the right side of the peaks. Symbols used: TG48:3, three double bonds present in TG48, i.e. tripalmitolein or tripalmitoleylglycerol; TG48:2, two double bonds present in TG48, i.e. dipalmitoleyl-palmitylglycerol; TG48:1, one double bond present in TG48, i.e. dipalmity-palmitoleylglycerol; TG48:0, saturated TG48, i.e. tripalmitate; In the presence of NH_4_^+1^, saturated TG48 was identified at m/z 824.8. Three unsaturated TG48 were identified at m/z 818.7, 820.8 and 822.8.

Mass spectrometry was employed for analyses of the forms of TG48 and it showed that indeed TG48:3, TG48:2, TG48:1 and TG48:0 species were all present in TG48 as shown in Fig. 2 and as predicted in Table 1. MS shows that the intensities of these four TG48 molecular species were significantly higher in CKD and HD patients than in healthy controls (Table 4). In fact, CKD patients had 7–13 times higher quantities of these 4 peaks and HD patients had 3–6 times higher quantities of the same peaks than those in healthy controls. Likewise, the relative quantities of TG48:3 and TG48:1 species, as judged by percentage of total TG48, were significantly different between CKD patients and controls (Table 4). The percentage of TG48:3 was significantly lower and TG48:1 was significantly higher in CKD patients than in controls. The percent composition of HD patients, though showing the same trend as those of CKD's, was not significantly different from controls. In CKD patients, the distribution of the TG48 species shows relatively more monounsaturated TG48:1 and relatively less polyunsaturated TG48:3 than controls. Therefore we can conclude that CKD patients have relatively more SFAs and relatively less un-SFAs than controls.

**Table 4:** Normalized intensities and percent composition of TG48 components in CKD, HD and controls.

Symbols	m/z	Mean ± SD	Patients	Mean ± SD	Patients
	with	Intensity x 10^−4^	vs	%	vs
	NH_4_^+1^		Controls		Controls
			p		p
		**CKD**		**CKD**	
CE-C4 (I.S.)	474.3				
TG48:3	818.8	151.8±40.5	<0.0005	11.17±2.26	<0.05
TG48:2	820.6	353.2±44.8	<0.0005	26.26±2.37	NS
TG48:1	822.6	576.8±38.4	<0.0005	43.01±3.33	<0.001
TG48:0	824.7	265.1±55.3	<0.0005	19.56±2.22	NS
		**HD**		**HD**	
TG48:3	818.8	77.1±52.6	<0.01	13.18±2.99	NS
TG48:2	820.6	177.5±133.2	<0.01	28.13±5.13	NS
TG48:1	822.6	277.9±230.9	<0.01	38.86±11.16	NS
TG48:0	824.7	122.6±90.5	<0.005	19.83±3.80	NS
		**Controls**		**Controls**	
TG48:3	818.8	22.5±14.6		16.01±3.55	
TG48:2	820.6	46.1±28.6		32.81±6.75	
TG48:1	822.6	42.1±20.5		32.65±2.92	
TG48:0	824.7	19.5±10.6		18.47±11.51	

Note: 4 CKD patients, 4 HD patients and 6 healthy controls were randomly selected for this study.

### Identification of the Fatty Acids in Various TG48 Forms by MS/MS

To further our research we determined what fatty acids were present in each form of these TG48 molecules. To characterize the fatty acids comprising TG48:3, TG48:2, TG48:1 and TG48:0, we performed MS/MS analysis of each TG48 form, corresponding to ammonium adducted ions, 818.7, 820.7, 822.7 and 824.7 m/z., respectively. The MS/MS fragmentation of TG results in the neutral losses of ammonia and a single fatty acid moiety and the formation of a positively charged diacylglycerol (DG) ions. Results are summarized in Table S1 (online table). To demonstrate an example of the analysis, the four ammonium adducted TG48 ions observed in the MS for patient A were each subjected to MS/MS fragmentation to determine their DG^+1^ fragments and constituent fatty acids. MS ion at m/z = 818.6, representing the TG48:3 molecules, was fragmented by MS/MS. Ammonia was lost to yield TG^+1^ at 801.7 m/z along with DG fragments and its product ions were determined. Seven observed DG ions (Fig. not shown) indicated that four different TG48:3 forms were present. LaOL (laurate/oleate/linoleate, C12:0/C18:1/C18:2) was confirmed based on the presence of ions 521.5 m/z, (DG_1,2_), 601.5 m/z (DG_2,3_) and 519.4 m/z (DG_1,3_). The DG ion profile of MPLn (myristate/palmitate/linoneneate, C14:0/C16:0/C18:3) was also observed to consist of DG_1,2_ at 523.5 m/z, DG_2,3_ at 573.5 m/z and DG_1,3_ at 545.5 m/z. Two other TG48:3 molecules were found to be present. They were MPoL (M/palmitoleate/L, C14:0/C16:1/C18:2), represented by DG_1,2_ at 521.5 m/z, DG_2,3_ at 573.5 m/z, DG_1,3_ at 547.5 m/z and PoPoPo (C16:1/C16:1/C16:1), in which all three DG forms produced an ion at 547.5 m/z. By the same approach, sample ion at 824.7 m/z (TG48:0) was fragmented by MS/MS. Ammonia was lost to yield TG^+1^ at 807.7 m/z along with three DG fragments, DG^+1^_1,2_ at 523.5 m/z, DG^+1^_2,3_ at 579.5 m/z and DG^+1^_1,3_ at 551.5 m/z. The ion at 551.5 was by far the most significant. This data indicated the presence of two different TG48:0 molecules, PPP (tripalmitate, C16:0/C16:0/C16:0) and MPS (M/P/stearate, C14:0/C16:0/C18:0). The PPP molecule was the predominant form, with all three of its DG forms generating the same single MS/MS fragment of 551.5. The MPS form was present at a lower level, and its MS/MS fragmentation produced all three DG ions of 523.5, 579.5 and 551.5, with the ions at 523.5 and 579.5 being less abundant, yet unique for the MPS form.

By the same approach, the fatty acids in TG48:2 (parent ion 820.7 m/z) and TG48:1 (parent ion 822.7 m/z) were also identified. The results are shown in Table S1 (online table). The fatty acids within the TG48 molecules were also determined for patients B, C and D and two control subjects E and F. The fatty acid composition of patient B was nearly identical to that of patient A. The fatty acid composition of patients C, D and control E was identical to that of patient A, and the fatty acid composition of control F was identical to that of patient B. All results are listed in Table S1 (online table).

It is interesting to note that the compositions of the fatty acids in TG48 in these six subjects are identical or nearly identical. Further exploration with additional patients or controls was thought unnecessary. We have now demonstrated that TG48 is a SFA-rich TG species in both patients and normal subjects. The difference between patients and controls is the quantity. Patients have many fold higher levels of SFAs than controls. Also CKD patients have lower percent polyunsaturated TG48:3 and higher percent monounsaturated TG48:1 than controls. When TGs' are partially hydrolysed in vivo, large amount of saturated DGs' are accumulated. Saturated DGs' and SFAs are inflammatory [18].

## Discussion

In this study, we have clearly established that TG48 is a SFA-rich TG species. Since SFAs are inflammatory [18], their increased levels reflect increased inflammation in renal patients. The association between SFAs and inflammation and their mechanisms has been well studied. SFAs inhibit the activation of insulin receptor substrate 1, which causes insulin resistance and contributes to metabolic syndrome [18]. This inhibition could occur in insulin-sensitive cells like white adipose tissue, muscle, heart and liver [23]. SFAs cause the activation of the mitogen-activated protein kinases and subsequent induction of inflammatory genes in white adipose tissue, immune cells, and mitotubes. SFAs decrease adiponectin production, which decreases the oxidation of glucose and fatty acids [18]. SFAs cause recruitment of immune cells, such as macrophages, neutrophils, and bone marrow-derived dendritic cells to white adipose tissue and muscle. Palmitate but not linoleate activates macrophages [24] which lead to the production of cytokine interleukin-6 in coronary artery endothelial cells and coronary artery smooth muscle cells [25]. Excess palmitate causes white adipose tissue dysregulation [26]. It also increases inflammation and apoptosis through oxidative or endoplasmic reticulum stress and generation of reactive oxygen species. Oleate co-supplementation blocks palmitate-mediated suppression of β-oxidation, insulin sensitivity, and DG accumulation. These experiments suggest that SFAs are inflammatory, while the unsaturated fatty acids are not [18]. Furthermore, higher polyunsaturated fatty acids, such as eicosapentaenoic and docosahexaenoic, have anti-inflammatory effects by inhibiting cyclooxygenase 2 and demonstrate the ability to reduce plasma concentrations of C-reactive protein (CRP), tumor necrotizing factor-α and interleukin-6 [27]. Interestingly, the carbon flux from a carbohydrate diet is converted to palmitate in the liver during endogenous lipogenesis [28]. Thus, a diet rich in carbohydrates can also be inflammatory.

As shown in Table 2, TG increased in patients with HD and CKD relative to levels in control subjects. TG48 also increased from controls to HD and to CKD. However, TG48/TG ratios (or TG48%/TG) are not the same for the three groups. TG48%/TG significantly (p<0.0005) increased from controls (1.7%±2.9%) to HD (4.2%±2.6%) and to CKD (5.2%±3.2%). This means that when TG is increased, TG48 is disproportionately increased. This results in the longer-chain TG species (TG50+TG52+TG54+TG56), increased less proportionally, because the sum of TG48+TG50+TG52+TG54+TG56 equals TG.

As shown by Byrdwell [21], the longer the carbon chain length of the fatty acids, the higher the unsaturation of the TG. Thus, TG48 possesses relatively higher number of SFAs than those of TG50, TG52, TG54 and TG56. Therefore, increasing TG results in increasing more of TG48, thus, increasing more of SFAs and concomitantly increasing inflammation.

We observed that subjects with increased TG without renal disease also have increased TG48 (unpublished data). So the presence of measurable TG48 by GC is not a unique marker for renal disease. It is actually a reflection of the increased SFAs, therefore a reflection of increased inflammation. Indeed, hypertriglyceridemia is also known to be associated with increased inflammation.

The presently established link between OS/inflammation and apoC-III bound to apoB-containing lipoproteins is further enhanced by the recently reported findings [13] that apoC-III, or its corresponding apoB-containing lipoproteins, were also proinflammatory as demonstrated by increasing endothelial cell expression of vascular and intercellular cell adhesion molecules and recruitment of monocytic cell. It is not surprising to see that TG48 is well correlated with apoC-III. Is there a cause- effect relationship between apoC-III and TG48? We are not certain. We may rationalize that when apoC-III is increased, TG would increase, since over expression of *apoC-III* gene could cause hypertriglyceridemia [29]. Our data indicate that when TG is increased, TG48 is increased more than the other TG molecular species. This implies that increased apoC-III could cause the increase of TG48 and thus increased levels of SFA. On the other hand, a diet rich in SFAs might increase TG. Would increased TG cause the increase of apoC-III? There might be such a possibility. However, more research is needed to answer this question unequivocally.

Inflammation causes the generation of reactive oxygen species [18]. This may be why inflammation is closely associated with OS and vice versa. Several studies support a link between OS and inflammation in atherogenesis [30], [31]. Our data showing that OS index R2 correlated with TG48 may support this notion.

A number of studies have suggested that increased OS in association with inflammation contributes significantly to the accelerated kidney dysfunction and its consequential cardiovascular morbidity and mortality [1]–[12]. Most of the previously described procedures to access OS are rather complicated and time consuming [2]–[7], [9], [12], [32]–[35]; however, our presently described method does not require treatment with chemical reagents, monoclonal antibodies, incubation, isolation of lipid classes or transesterification and therefore, provide a direct and very useful simplification. The main advantage of the GC method besides simplicity and speed is high reproducibility. R2 values have shown that a combination of OS and the levels of apoC-III in a binary system predict the risk for atherosclerosis in normolipidemic and hypertriglyceridemic subjects [36]. Cholesteryl ester ratio method has also been successfully used to monitor the delay of OS recovery in patients with diabetic ketoacidosis after correction of ketoacidosis with insulin [37]. Inflammation levels have been most frequently measured by CRP [38] and interleukin-6 [25], [27]. Both of these proteins are the results of inflammation. In contrast, SFAs measured by TG48 are the causes of inflammation.

Our data show that the inflammation measured by TG48 is correlated with TG, not CE. Since TG48 is part of the total TG, in general, the higher the TG, the higher the TG48, thus the higher the SFA and the higher the inflammation. Indeed, higher TG is associated with higher OS, as also shown by the CE-ratio method, but TG alone does not represent TG48 because the TG48/TG ratio is not a constant among different subjects.

Abnormalities of lipid and apolipoprotein profiles of CKD and HD patients confirmed and extended the results of our previous studies [15], [16]. Moreover, based on correlation coefficients between R2 and especially, TG48 and lipid, apolipoprotein, and lipoprotein-cholesterol levels, it was established that inflammation is highly correlated with the concentrations of TG and VLDL-C, moderately correlated with apoC-III bound to apoB-containing lipoproteins, and weakly with apoC-III, but not with TC, LDL-C nor with HDL-C. These results suggest that the high inflammation of renal patients stems more from TG-rich lipoproteins, not CE-rich apoB lipoproteins. The highly selective correlation of inflammation with TG or apoC-III-containing apoB lipoproteins, but not with cholesterol-rich apoB lipoproteins, was confirmed by the results of multivariate regression analysis showing a stronger relationship between TG48 and VLDL-C over LDL-C (15 times stronger for VLDL-C than that for LDL-C as shown by the equation #1).

Results from our study and those of other laboratories have already shown the significant role played by apoC-III bound to apoB-containing lipoproteins in the formation and progression of atherosclerotic lesion [39]. Specifically, renal insufficiency resulting in elevated apoC-III, or LpB:C-III, presumably via insulin resistance triggers the chain of inflammatory events [40]. Taken together, findings of the present study suggest that TG-rich apoC-III-containing apoB lipoproteins are linked to inflammation, while TC-rich apoB lipoproteins are not. It remains to be determined whether there are differences in the degree of inflammation between apolipoprotein-defined apoC-III-containing lipoproteins, including LpB:C, LpB:C:E and LpA-II:B:C:D:E particles.

The use of the TG48 marker revealed that HD patients had significantly lower inflammation and partially reduced levels of lipoprotein variables than CKD patients, suggesting a beneficial effect of haemodialysis that alleviates the uremic intoxication but cannot eliminate the underlying metabolic disturbance that persists during dialysis.

It has been observed that increased OS is already prevalent in CKD patients before dialysis [5], [41]. Our findings on increased OS/inflammation in renal patients are in agreement with the literature reports [2]–[12], [33]. This is the first report using TG48 for measuring SFAs and inflammation. This is also the first report to relate SFAs and inflammation with lipids and lipoproteins, and particularly, with apoC-III and apoC-III-containing apoB lipoproteins.

### Conclusions

The major finding of this study is the recognition of TG48 as a new inflammatory factor characterized by the occurrence of inflammatory saturated fatty acids. The levels of TG48 had been found to be elevated in patients with chronic renal disease in comparison with those of normolipidemic subjects. The TG48 levels were correlated with the levels of TG, VLDL-C, apoC-III and apoC-III bound to apoB-containing lipoprotein subclasses, but not with TC, LDL-C and HDL-C in kidney patients. Furthermore TG48 is also correlated with oxidative stress. The well established dual role of apoC-III in inflammation and atherogenesis in combination with oxidative stress and inflammatory TG48 should be considered as severe risk factors for atherogenesis in chronic kidney disease. Further studies are needed to establish the occurrence of similar risk factors for atherogenesis in other dyslipoproteinemics.

## Supporting Information

Table S1 **Triacylglycerols TG48 Predicted by Analysis of Fragments Generated by MS/MS.** Abbreviations used: DG, diglycerides; TG, triacylglycerols; La, laurate; M, myristate; P, palmitate; S, stearate; O, oleate; Po, pamitoleate; L, linoleate; ^a^in small quanity.
(DOC)

## References

[1] Foley RN, Parfrey PS, Sarnak MJ (1998). Clinical epidemiology of cardiovascular disease in chronic renal disease.. Am J Kidney Dis.

[2] Soejima A, Kaneda F, Manno S, Matsuzawa N, Kouji H (2002). Useful markers for detecting decreased serum antioxidant activity in hemodialysis patients.. Am J Kidney Dis.

[3] Kumano K, Yokota S, Go M, Suyama K, Sakai T (1992). Quantitative and qualitative changes of serum albumin in CAPD patients.. Adv Perit Dial.

[4] Diepeveen SHA, Verhoeven GWHE, van der Palen J, Dikkeschei LD, van Tits LJ (2005). Effects of atorvastatin and vitamin E on lipoproteins and oxidative stress in dialysis patients: a randomised-controlled trial.. J Intern Med.

[5] Oberg BP, McMenamin E, Lucas FL, McMonagle E, Morrow J (2004). Increased prevalence of oxidant stress and inflammation in patients with moderate to severe chronic kidney disease.. Kidney Int.

[6] Terawaki H, Yoshimura K, Hasegawa T, Matsuyama Y, Negawa T (2004). Oxidative stress is enhanced in correlation with renal dysfunction: examination with the redox state of albumin.. Kidney Int.

[7] Boaz M, Matas Z, Biro A, Katzir Z, Green M (1999). Serum malondialdehyde and prevalent cardiovascular disease in hemodialysis.. Kidney Int.

[8] Handelman GJ, Walter MF, Adhikarla R, Gross J, Dallal GE (2001). Elevated plasma F2-isoprostanes in patients on long-term hemodialysis.. Kidney Int.

[9] Boulanger E, Moranne O, Wautier MP, Witko-Sarsat V, Descamps-Latscha B (2006). Changes in glycation and oxidation markers in patients starting peritoneal dialysis: a pilot study.. Perit Dial Int.

[10] Taki K, Takayama F, Tsuruta Y, Niwa T (2006). Oxidative stress, advanced glycation end product, and coronary artery calcification in hemodialysis patients.. Kidney Int.

[11] Dogra G, Irish A, Chan D, Watts G (2006). Insulin resistance, inflammation, and blood pressure determine vascular dysfunction in CKD.. Am J Kidney Dis.

[12] Annuk M, Soveri I, Zilmer M, Lind L, Hulthe J (2005). Endothelial function, CRP and oxidative stress in chronic kidney disease.. J Nephrol.

[13] Kawakami A, Aikawa M, Alcaide P, Luscinskas FW, Libby P (2006). Apolipoprotein CIII induces expression of vascular cell adhesion molecule-1 in vascular endothelial cells and increases adhesion of monocytic cells.. Circulation.

[14] Lee DM (1999). A simple and sensitive method in using the ratios of cholesteryl ester molecular species as indexes of oxidative stress in plasma and lipoprotein fractions.. Atherosclerosis.

[15] Attman P-O, Alaupovic P (1991). Lipid and apolipoprotein profiles of uremic dyslipoproteinemia - relation to renal function and dialysis.. Nephron.

[16] Alaupovic P, Attman P-O, Knight-Gibson C, Mulec H, Weiss L (2006). Effect of fluvastatin on apolipoprotein-defined lipoprotein subclasses in patients with chronic renal insufficiency.. Kidney Int.

[17] Kuksis A, Myher JJ, Marai L, Geher K (1975). Determination of plasma lipid profiles by automated gas chromatography and computerized data analysis.. J Chromatogr Sci.

[18] Kennedy A, Martinez K, Chuang CC, LaPoint K, McIntosh M (2009). Saturated fatty acid-mediated inflammation and insulin resistance in adipose tissue: mechanisms of action and implications.. J Nutr.

[19] Liebisch G, Binder M, Schifferer R, Langmann T, Schulz B (2006). High throughput quantification of cholesterol and cholesteryl ester by electrospray ionization tandem mass spectrometry (ESI-MS/MS).. Biochim Biophys Acta.

[20] McAnoy AM, Wu CC, Murphy RC (2005). Direct qualitative analysis of triacylglycerols by electrospray mass spectrometry using a linear ion trap.. J Am Soc Mass Spectrom.

[21] Byrdwell WC (2005). The bottom-up solution to the triacylglycerol lipidome using atmospheric pressure chemical ionization mass spectrometry.. Lipids.

[22] Benjamini Y, Drai D, Elmer G, Kafkafi N, Golani I (1995). Controlling the false discovery rate: a practical and powerful approach to multiple testing. J Roy. Statist Soc.. Ser B.

[23] Guilherme A, Virbasius JV, Puri V, Czech MP (2008). Adipocyte dysfunctions linking obesity to insulin resistance and type 2 diabetes.. Nat Rev Mol Cell Biol.

[24] Suganami T, Tanimoto-Koyama K, Nishida J, Itoh M, Yuan X (2007). Role of the Toll-like receptor 4/NF-kappaB pathway in saturated fatty acid-induced inflammatory changes in the interaction between adipocytes and macrophages.. Arterioscler Thromb Vasc Biol.

[25] Staiger H, Staiger K, Stefan N, Wahl HG, Machicao F (2004). Palmitate-induced interleukin-6 expression in human coronary artery endothelial cells.. Diabetes.

[26] Takahashi K, Yamaguchi S, Shimoyama T, Seki H, Miyokawa K (2008). JNK- and IkappaB-dependent pathways regulate MCP-1 but not adiponectin release from artificially hypertrophied 3T3-L1 adipocytes preloaded with palmitate in vitro.. Am J Physiol Endocrinol Metab.

[27] Simopoulos AP (2002). Omega-3 fatty acids in inflammation and autoimmune diseases.. J Am Coll Nutr.

[28] Okada T, Furuhashi N, Kuromori Y, Miyashita M, Iwata F (2005). Plasma palmitoleic acid content and obesity in children.. Am J Clin Nutr.

[29] Ito Y, Azrolan N, O'Connell A, Walsh A, Breslow JL (1990). Hypertriglyceridemia as a result of human apo-CIII gene expression in transgenic mice.. Science.

[30] Singh U, Devaraj S, Jialal I (2005). Vitamin E, oxidative stress, and inflammation.. Annu Rev Nutr.

[31] Ross R (1999). Atherosclerosis is an inflammatory disease.. Am Heart J.

[32] Winterbourn CC, Buss IH (1999). Protein carbonyl measurement by enzyme-linked immunosorbent assay.. Methods Enzymol.

[33] Himmelfarb J, McMonagle E, McMenamin E (2000). Plasma protein thiol oxidation and carbonyl formation in chronic renal failure.. Kidney Int.

[34] Sogami M, Era S, Nagaoka S, Kuwata K, Kida K (1985). High-performance liquid chromatographic studies on non-mercapt in equilibrium with mercapt conversion of human serum albumin. II.. J Chromatogr.

[35] Gutteridge JMC, Halliwell B (1990). The measurement and mechanism of lipid peroxidation in biological systems.. Trends Biochem Sci.

[36] Lee DM, Purdie N, Gibson C (2000). Oxidative stress and apolipoprotein C-III in binary system predicts the levels of risk for arteriosclerosis in normolipidemic and hypertriglyceridemic subjects.. Atherosclerosis.

[37] Lee DM, Hoffman WH, Carl GF, Khichi M, Cornwell PE (2002). Lipid peroxidation and antioxidant vitamins prior to, during, and after correction of diabetic ketoacidosis.. J Diabet Complications.

[38] Bowden RG, Wilson RL (2010). Malnutrition, inflammation, and lipids in a cohort of dialysis patients.. Postgrad. Med.

[39] Alaupovic P, Mack WJ, Knight-Gibson C, Hodis HN (1997). The role of triglyceride-rich lipoprotein families in the progression of atherosclerotic lesions as determined by sequential coronary angiography from a controlled clinical trial.. Arterioscler Thromb Vasc Biol.

[40] Attman PO, Samuelsson O, Alaupovic P (2011). The effect of decreasing renal function on lipoprotein profiles.. Nephrol Dial Transplant.

[41] Siems W, Quast S, Carluccio F, Wiswedel I, Hirsch D (2002). Oxidative stress in chronic renal failure as a cardiovascular risk factor.. Clin Nephrol.

