# Supplementary material for: Oxidative Stress and Inflammation in Renal Patients and Healthy Subjects
Source: PLoS One. 2011 Jul 28;6(7):e22360. doi: 10.1371/journal.pone.0022360 (PMC3145638; doi:10.1371/journal.pone.0022360)
Supplement: Table S1 — Triacylglycerols TG48 Predicted by Analysis of Fragments Generated by MS/MS. Abbreviations used: DG, diglycerides; TG, triacylglycerols; La, laurate; M, myristate; P, palmitate; S, stearate; O, oleate; Po, pamitoleate; L, linoleate; ain small quanity. (DOC) [file pone.0022360.s001.doc]

**Table S1**

**Triacylglycerols TG48 Predicted by Analysis of Fragments Generated by MS/MS**

**Fragmentation of Detected DG’s and TG^+1^ Predicted Carbon atom number Types of**

**Subject Parent ion TG^+1^ DG1,2 DG2,3 DG1,3 Possible TG and unsaturation TG48**

| Patient A | 818.7 m/z | 801.7 | 521.5 | 601.5 | 519.4 | LaOL | C12:0/C18:1/C18:2 | TG48:3 |
| --- | --- | --- | --- | --- | --- | --- | --- | --- |
|  |  |  | 523.5 | 573.5 | 545.5 | MPLn | C14:0/C16:0/C18:3 | TG48:3 |
|  |  |  | 521.5 | 573.5 | 547.5 | MPoL | C14:0/C16:1/C18:2 | TG48:3 |
|  |  |  |  | 547.5 |  | PoPoPo | C16:1/C16:1/C16:1 | TG48:3 |
|  |  |  |  |  |  |  |  |  |
|  | 820.7 m/z | 803.8 | 523.5 | 575.5 | 547.5 | MPL | C14:0/C16:0/C18:2 | TG48:2 |
|  |  |  | 521.5 | 575.5 | 549.5 | MPoO | C14:0/C16:1/C18:1 | TG48:2 |
|  |  |  | 603.5 | 521.5 |  | OOLa | C18:1/C18:1/C12:0 | TG48:2 |
|  |  |  |  |  |  |  |  |  |
|  | 822.7 m/z | 805.8 | 523.5 | 577.5 | 549.5 | MPO | C14:0/C16:0/C18:1 | TG48:1 |
|  |  |  | 551.5 | 549.5 |  | PPPo | C16:0/C16:0/C16:1 | TG48:1 |
|  |  |  |  |  |  |  |  |  |
|  | 824.74 m/z | 807.7 |  | 551.5 |  | PPP | C16:0/C16:0/C16:0 | TG48:0 |
|  |  |  | 523.5 | 579.5 | 551.5 | MPS^a^ | C14:0/C16:0/C18:0 | TG48:0 |
|  |  |  |  |  |  |  |  |  |
| Patient B | 818.7 m/z | 801.7 | 521.5 | 601.5 | 519.4 | LaOL | C12:0/C18:1/C18:2 | TG48:3 |
|  |  |  | 523.5 | 573.5 | 545.5 | MPLn | C14:0/C16:0/C18:3 | TG48:3 |
|  |  |  | 521.5 | 573.5 | 547.5 | MPoL | C14:0/C16:1/C18:2 | TG48:3 |
|  |  |  |  | 547.5 |  | PoPoPo | C16:1/C16:1/C16:1 | TG48:3 |
|  |  |  |  |  |  |  |  |  |
|  | 820.7 m/z | 803.7 | 523.5 | 575.5 | 547.5 | MPL | C14:0/C16:0/C18:2 | TG48:2 |
|  |  |  | 603.5 | 521.5 |  | OOLa | C18:1/C18:1/C12:0 | TG48:2 |
|  |  |  | 547.5 | 549.5 |  | PoPoP | C16:1/C16:1/C16:0 | TG48:2 |
|  |  |  | 521.5 | 575.5 | 549.5 | MPoO | C14:0/C16:1/C18:1 | TG48:2 |
|  |  |  |  |  |  |  |  |  |
|  | 822.7 m/z | 805.8 | 523.5 | 577.5 | 549.5 | MPO | C14:0/C16:0/C18:1 | TG48:1 |
|  |  |  | 551.5 | 549.5 |  | PPPo | C16:0/C16:0/C16:1 | TG48:1 |
|  |  |  |  |  |  |  |  |  |
|  | 824.74 m/z | 807.8 |  | 551.5 |  | PPP | C16:0/C16:0/C16:0 | TG48:0 |
|  |  |  |  |  |  |  |  |  |
| Patient C | 818.7 m/z | 801.7 | 521.5 | 601.5 | 519.4 | LaOL | C12:0/C18:1/C18:2 | TG48:3 |
|  |  |  | 523.5 | 573.5 | 545.5 | MPLn | C14:0/C16:0/C18:3 | TG48:3 |
|  |  |  | 521.5 | 573.5 | 547.5 | MPoL | C14:0/C16:1/C18:2 | TG48:3 |
|  |  |  |  | 547.5 |  | PoPoPo | C16:1/C16:1/C16:1 | TG48:3 |
|  |  |  |  |  |  |  |  |  |
|  | 820.7 m/z | 803.8 | 523.5 | 575.5 | 547.5 | MPL | C14:0/C16:0/C18:2 | TG48:2 |
|  |  |  | 521.5 | 575.5 | 549.5 | MPoO | C14:0/C16:1/C18:1 | TG48:2 |
|  |  |  | 603.5 | 521.5 |  | OOLa | C18:1/C18:1/C12:0 | TG48:2 |
|  |  |  |  |  |  |  |  |  |
|  | 822.7 m/z | 805.8 | 523.5 | 577.5 | 549.5 | MPO | C14:0/C16:0/C18:1 | TG48:1 |
|  |  |  | 551.5 | 549.5 |  | PPPo | C16:0/C16:0/C16:1 | TG48:1 |
|  |  |  |  |  |  |  |  |  |
|  | 824.74 m/z | 807.7 |  | 551.5 |  | PPP | C16:0/C16:0/C16:0 | TG48:0 |
|  |  |  | 523.5 | 579.5 | 551.5 | MPS^a^ | C14:0/C16:0/C18:0 | TG48:0 |
| Patient D Results were the same as Patient A  Control E Results were the same as Patient A  Control F Results were the same as Patient B | | | | | | | |  |
| Abbr used: DG, diglycerides; TG, triacylglycerols; La, laurate; M, myristate; P, palmitate; S, stearate; O, oleate; Po, pamitoleate; | | | | | | | | |
|  | L, linoleate; Ln, linolineate; ^a^in small quantity | | |  |  |  |  |  |
